# Comorbidity and survival of Danish breast cancer patients from 1995 to 2005

**DOI:** 10.1038/sj.bjc.6603717

**Published:** 2007-04-03

**Authors:** D P Cronin-Fenton, M Nørgaard, J Jacobsen, J P Garne, M Ewertz, T L Lash, H T Sørensen

**Affiliations:** 1 Department of Clinical Epidemiology, Aarhus University Hospital, Ole Worms Allé 1150, Aarhus C 8000, Denmark; 2 Department of Surgical Oncology, Aalborg University Hospital, Aalborg, Denmark; 3 Department of Epidemiology, School of Public Health, Boston University, 715 Albany Street, TE3, Boston, MA 02118, USA; 4 Department of Oncology, Aalborg Hospital, Aalborg, Denmark

**Keywords:** breast cancer, survival, Charlson comorbidity score, population-based

## Abstract

Comorbid diseases can affect breast cancer prognosis. We conducted a population-based study of Danish women diagnosed with a first primary breast cancer from 1995 to 2005 (*n*=9300), using hospital discharge registry data to quantify comorbidities by Charlson score. We examined the influence of comorbidities on survival, and quantified their impact on relative mortality rates. The prevalence of patients with a Charlson score=‘0’ fell from 86 to 81%, with an increase in those with Charlson score=‘1–2’ from 13 to 16%, and score=‘3+’ from 1 to 2%. One- and five-year survival for patients with Charlson score=‘0’ and ‘1–2’ was better for those diagnosed in 1998–2000 than in 1995–1997. Overall, patients diagnosed in 2001–2004 (mortality ratio (MR)=0.80, 95% CI=0.68–0.95) and 1998–2000 (MR=0.92, 95% CI=0.78–1.09) had lower 1-year age-adjusted mortality compared to those diagnosed in 1995–1997 (reference period). Patients with Charlson scores ‘1–2’ and ‘3+’ had higher age-adjusted 1-year mortality than those with a Charlson score=‘0’ in each time period (2001–2004: MR_‘1–2’_=1.76, 95% CI=1.35–2.30, and MR_‘3+’_=3.78, 95% CI=2.51–5.68; and 1998–2000: MR_‘1–2’_=1.60, 95% CI=1.36–1.88 and MR_‘3+’_=2.34, 95% CI=1.65–3.33). Similar findings were observed for 5-year age-adjusted mortality. Additional analyses, adjusted for stage, indicated that confounding by stage could not explain these findings. Despite continued improvements in breast cancer survival, we found a trend of poorer survival among breast cancer patients with severe comorbidities even after adjusting for age and stage. Such poorer survival is an important public health concern and can be expected to worsen as the population ages.

Breast cancer accounts for a substantial proportion of the cancer burden, with over 1 000 000 new cases diagnosed and over 400 000 breast cancer-related deaths worldwide each year (Parkin *et al*, 2005). Denmark has the highest age-standardised breast cancer incidence rate, and one of the highest mortality rates in the world (Globocan, 2006; National Board of Health, 2006).

Over 50% of newly diagnosed breast cancers occur in women aged 60 years or older. Many of these women have coexistent diseases (comorbidities) at the time of their breast cancer diagnosis, which can substantially influence their diagnostic work-up, treatment options, and survival (West *et al*, 1996; Potosky *et al*, 2002; Harlan *et al*, 2003a; Louwman *et al*, 2005). The presence of comorbidities at diagnosis can have a negative impact on prognosis and survival (West *et al*, 1996; Cronin *et al*, 2005), and two small studies have indicated that breast cancer patients with comorbid conditions have lower survival compared to those without comorbidities (Charlson *et al*, 1987; West *et al*, 1996).

The change in population demographics, in terms of population aging (Coebergh, 1996), will result in an increased proportion of elderly cancer patients, many of whom present with comorbid diseases. A study by Louwman *et al* (2005) indicated that 10% of patients aged less than 50 years had comorbid conditions compared to 55% of patients aged over 80 years.

We present a population-based study of the impact of comorbidity (as measured by the Charlson comorbidity score, originally validated using 10-year survival in breast cancer patients) on breast cancer survival and mortality in a Danish population from 1995 to 2005 (Charlson *et al*, 1987). We examine trends in breast cancer survival and mortality over three time periods.

## MATERIALS AND METHODS

### Study population

We identified all patients with a diagnosis of breast cancer (ICD-8 code 174.xx and ICD-10 code C50.x) from 1 January 1995 through 31 March 2004, using the hospital discharge registries of four Danish counties; North Jutland, Aarhus, Viborg, and Ringkjøbing. In Denmark, all health-related services are registered to individual patients by use of their civil personal registry number (CPR), assigned to all Danish citizens since 1968, which denotes gender and date of birth. This unique CPR number facilitates linkage between population-based registries (Gaist *et al*, 1997). The registries include all non-psychiatric hospital admissions (since 1977) and outpatient hospital visits (since 1995). Information is recorded immediately after discharge and includes CPR number, dates of admission and discharge, and up to 20 discharge diagnoses (Andersen *et al*, 1999). Diagnoses are classified according to the International Classification of Diseases (ICD), 8th revision until the end of 1993 and the 10th revision thereafter.

### Comorbidities at diagnosis

We included information on comorbidities up to 10 years before breast cancer diagnosis. Comorbidities were identified using the hospital discharge registries of each county and were categorised using the Charlson index. The index comprises 19 conditions, each weighted according to its potential to influence mortality (Charlson *et al*, 1987). We used Deyo's adaptation of the Charlson comorbidity score, which adapts the Charlson clinical comorbidity index for research relying on ICD-10 codes (Deyo *et al*, 1992). Breast cancer diagnoses were not included when computing the index. We grouped patients according to a Charlson score of ‘0’, ‘1–2’, or ‘3+’, groupings which translate into ‘mild’, ‘moderate’, and ‘severe’ illness (Charlson *et al*, 1987).

### Stage at diagnosis

Information on stage is not included in the hospital discharge registries. To obtain information on breast cancer stage at diagnosis, we used the CPR number to link to the Danish Cancer Registry. The Danish Cancer Registry is a population-based nationwide registry with data on incident cases of cancer in Denmark since 1943, including civil registration number, method of verification of the cancer, stage, and residence at date of cancer diagnosis. The registry receives notifications from hospital departments, institutes of forensic medicine, general practitioners, and practising specialists The registry is not, however, entirely up to date. Data are complete through 2002.

### Vital status

We linked members of the study cohort via their CPR number to the Danish Civil Registry to obtain vital status. The Danish Civil Registry is updated daily and has maintained records on vital status, date of death, and the residence of all Danish citizens since 1 April 1968. Follow-up was through patient date of death or 31 January 2005, whichever occurred first.

### Statistical analyses

We present distribution frequencies (numbers and percentages) of breast cancer patients in each Charlson score category for each period of diagnosis. We plotted Kaplan–Meier curves for breast cancer patients according to the periods of diagnosis – 1995–1997, 1998–2000 and 2001–2004, age, and comorbidity categories. All time periods were 36 months long, except for the last time period, which included 39 months. We calculated survival at 1 and 5 years by product limit methods for each group, except for 5-year survival for patients diagnosed from 2001 to 2004. We used Cox proportional hazards regression analysis to compute 1- and 5-year crude and age-adjusted hazard ratios as a measure of relative mortality to assess the association of comorbidity with relative mortality using the Charlson score ‘0’ as the reference category in each time period. For the patients with available stage information, we carried out a subanalysis adjusting mortality ratios for both age and stage.

## RESULTS

Table 1 illustrates the number and percentage of patients for each Charlson comorbidity score by diagnostic period. A total of 9300 breast cancer cases were included, 2819 diagnosed in 1995–1997, 3003 diagnosed in 1998–2000, and 3478 diagnosed from 2001–2004. The prevalence of patients with Charlson score ‘0’ fell from 86 to 81% over the time period, with corresponding increases in the prevalence of patients with Charlson scores ‘1–2’ (3% increase) and ‘3+’ (1% increase). For all three time periods, median age at diagnosis did not change for patients with a Charlson score ‘0’ (59 out of 60 years) or a Charlson score ‘1–2’ (72 out of 73 years). Among patients with a Charlson score ‘3+’, however, there was a gradual increase in the median age at diagnosis with each diagnostic period from age 72 to 76 years in the respective calendar year categories. The highest number of patients resided in Aarhus and North-Jutland Counties because of population demographics. There was a sharp increase in the proportion of patients with a Charlson score ‘1–2’ in Ringkojbing County from 1995–1997 to 1998–2000 from 8 to 14%, and then to 17% in 2001–2004. There was an increase in the proportion of breast cancers in the latest period in all counties.

**Figure 1:**
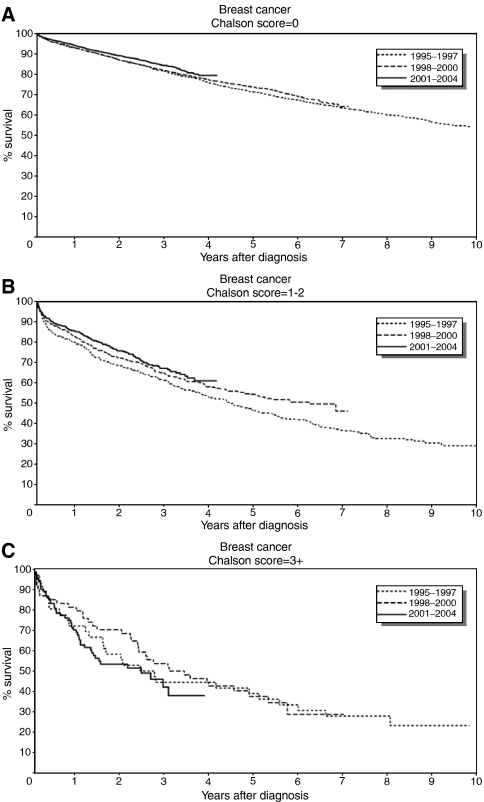
Kaplan–Meier survival curves for patients with breast cancer in Denmark for three time periods for (**A**) Charlson score=0, (**B**) Charlson score=1–2, and (**C**) Charlson score=3+.

Figure 1 shows survival curves for breast cancer patients diagnosed in each period according to Charlson comorbidity score category. A slightly better survival was noted among patients with Charlson score ‘0’ diagnosed from 2001 to 2004, and with Charlson score ‘1–2’ diagnosed from 1998 to 2000 and from 2001 to 2004 (Figure 1A and B). There was no improvement in survival for patients with Charlson scores ‘3+’ over the time periods (Figure 1C).

Table 2 outlines the cumulative proportion of patients surviving at 1-year and 5-years as well as the crude and adjusted relative mortality rates comparing patients with positive Charlson comorbidity scores to those with Charlson score ‘0’. One-year survival of patients with Charlson score ‘0’ was similar for all three diagnostic periods. Five-year survival was slightly better in patients diagnosed in 1998–2000 (70%, 95% CI=68–72%) compared to those diagnosed in 1995–1997 (68%, 95% CI=66–69%). One-year survival was also better among patients with Charlson score ‘1–2’ diagnosed in 2001–2004 compared to those diagnosed during the earlier periods (85 compared to 81% (1998–2000) and 79% (1995–1997)). Among patients with Charlson score ‘3+’, 1-year survival was lowest in those diagnosed from 2001–2004 (67%, 95% CI=55–76%), compared to those diagnosed in 1998–2000 (80%, 95% CI=66–88%) and in 1995–1997 (72%, 95% CI=55–84%).

Patients with non-zero comorbidity scores had poorer survival than those with Charlson score ‘0’ in each diagnostic period. One-year relative mortality among patients with Charlson score ‘1–2’ was higher than that of their Charlson score ‘0’ counterparts; mortality ratio (MR)_1995–1997_=2.43 (95% CI=1.84–3.21), MR_1998–2000_=2.01, 95% CI=1.53–2.63, and MR_2001–2004_=1.76, 95% CI=1.35–2.30. Five-year relative mortality was also higher for patients with Charlson score ‘1–2’ compared to those with Charlson score ‘0’; MR_1995–1997_=1.89, 95% CI=1.61–2.33, and MR_1998–2000_=1.60, 95% CI=1.36–1.88. Among patients with Charlson score ‘3+’, 1-year relative mortality was 2–3 times that of Charlson score ‘0’ patients in each time period; MR_1995–1997_=3.21, 95% CI=1.69–6.08, MR_1998–2000_=2.25, 95% CI=1.22–4.15, and MR_2001–2004_=3.78, 95% CI=2.51–5.68. A similar effect of higher mortality among patients with a Charlson score of ‘3+’ was seen for 5-year mortality; MR_1995–1997_=2.43 (95% CI=1.59–3.72) and MR_1998–2000_=2.34 (95% CI=1.65–3.33).

Although we observed little difference in the proportion of patients alive 1-year post-diagnosis, overall, relative mortality was lower for patients diagnosed between 2001 and 2004 after adjusting for age and comorbidity with a 1-year MR=0.80 (95% CI=0.68–0.95) for 2001–2004 compared to earlier periods and a 5-year MR=0.88 (95% CI=0.80–0.97) for 1998–2000 (Table 3).

Stage information was available on 83% of the patients, amounting to 7702 cases. On these patients, we carried out a subanalysis (Table 4). For each Charlson score, there was a decrease in local, distant, and unstaged disease and an increase in regional stage over time. The prevalence of patients with unstaged disease was higher among those with Charlson scores ‘1–2’ and ‘3+’ compared to those with Charlson score ‘0’. After adjusting for age and stage, 1-year relative mortality among patients with Charlson score ‘1–2’ was approximately twice that of Charlson score ‘0’ patients; MR_1995–1997_=2.67, 95% CI=1.92–3.72, MR_1998–2000_=2.14, 95% CI=1.54–2.96, and MR_2001–2004_=1.91, 95% CI=1.36–2.69. Five-year relative mortality was also higher for patients with a Charlson score ‘1–2’ compared to those with a Charlson score ‘0’ in each period; MR_1995–1997_=1.92, 95% CI=1.59–2.32 and MR_1998–2000_=1.63, 95% CI=1.35–1.98. Among patients with a Charlson score ‘3+’, 1-year relative mortality was at least three times that of Charlson score ‘0’ patients; MR_1995–1997_=3.33, 95% CI=1.62–6.87, MR_1998–2000_=4.40, 95% CI=2.11–9.14, and MR_2001–2004_=4.45, 95% CI=2.58–7.66. Patients with a Charlson score of ‘3+’ had higher 5-year mortality; MR_1995–1997_=2.58, 95% CI=1.56–4.25 and MR_1998–2000_=3.78, 95% CI=2.51–5.69.

## DISCUSSION

There are two key findings of this population-based study. First, we note a trend of poorer survival from 1995 through 2005 among breast cancer patients with comorbid diseases. To our knowledge, this has not been reported previously. The second key finding is that the overall prognosis for breast cancer patients improved over time after adjusting for age and comorbidity, similar to trends in other populations, where breast cancer survival has improved over time (Sant *et al*, 2001; Althuis *et al*, 2005; Olsen *et al*, 2005).

Breast cancer survival can be improved via earlier and more adequate diagnosis, and up-to-date guideline concordant treatment. Over the period studied, adjuvant breast cancer treatment adhered to the guidelines of the Danish Breast Cancer Cooperative Group (www.dbcg.dk). There was no population-based mammography screening in the study area. Moreover, there were no major changes to diagnostic techniques in the study population, although the sentinel lymph node biopsy (SLNB) was increasingly used to spare women, with no sign of axillary disease, a full dissection. Such increased use of SLNB may contribute to the increase in regional stage over time for each Charlson category, and may have caused some level of disease upstaging consistent with trends in other countries (Schouten *et al*, 2002) (Cronin-Fenton *et al*, 2007, manuscript in submission). The rising incidence rates of breast carcinoma with micro-metastatic lymph node involvement.

Breast cancer survival has been extensively studied (Bergman *et al*, 1991a, 1991b; Ewertz, 1993; Carmichael *et al*, 2004; Althuis *et al*, 2005; Grant, 2005; Parkin *et al*, 2005); however, a relative paucity of research has investigated the effect of comorbidity on breast cancer and cancer survival generally (Bergman *et al*, 1991a; West *et al*, 1996; Yancik *et al*, 2001). Our study extends the research from several studies in western populations that indicate a negative impact of comorbidity on cancer survival (Charlson *et al*, 1987; West *et al*, 1996; Cronin *et al*, 2005). Our findings compare to those of Charlson *et al* (1987) and West *et al* (1996), highlighting the increased risk of mortality associated with mild (Charlson score ‘1–2’) and severe comorbidity (Charlson score ‘3+’) compared to little/no comorbidity (Charlson score ‘0’). However, like the West study, we assessed all-cause mortality rather than mortality from causes other than breast cancer as per the Charlson paper. Thus cancer, or its treatment, may accelerate the course of other pathological conditions, resulting in poorer survival.

Studies have indicated that patients with comorbidities are often (West *et al*, 1996; Potosky *et al*, 2002; Harlan *et al*, 2003a; Louwman *et al*, 2005), but not always (Lash *et al*, 2003), less likely to receive appropriate treatment. Lower survival of patients with comorbidities may be attributable to physician or patient preferences to forego the potential toxicities of cancer-directed therapy, which could further compromise their health and quality of life. Although less treatment of these patients may have a negative impact on their survival – thus our observed poorer survival among patients with comorbidities compared to those with a Charlson score of zero – it is unlikely to influence their survival over time as there has been little change to breast cancer therapy from 1995 to 2004 (www.dbcg.dk).

An interesting finding of our study is the increase in median age of patients with severe comorbidities. However, the prevalence of patients with a Charlson comorbidity score increased only slightly over the period of diagnosis, largely driven by an increased prevalence of patients with Charlson score ‘1–2’ rather than those with score ‘3+’. The trend of higher mortality among patients with comorbidities remained even after adjusting for age and stage. Breast cancer therapy (and cancer therapy in general) differs according to patient age and menopausal status; older women can be less likely than younger women to receive definitive care for newly diagnosed breast cancer (Bergman *et al*, 1991a; Havlik *et al*, 1994; Hurria *et al*, 2003; Harlan *et al*, 2003b; Janssen-Heijnen *et al*, 2005; Louwman *et al*, 2005). In Denmark, chemotherapy is not recommended as a standard treatment for hormone receptor-negative patients over the age of 70 years. Furthermore, adjuvant therapy is rarely given to patients over the age of 75 years at diagnosis.

We note a higher prevalence of patients with unstaged disease had Charlson scores of ‘1–2’ or ‘3+’. This may suggest that the presence of comorbidity at diagnosis either prevented a full diagnostic work-up or the assignment of an appropriate stage. Moreover, as anticancer therapy is directed by stage at diagnosis, patients with unstaged disease may have received suboptimal cancer treatment. These factors are likely to have exacerbated the prognosis of these patients.

Our findings indicate an increase in the proportion of patients with a comorbidity score between the first and last time periods. This may be attributable to more complete recording of comorbid disease in the hospital discharge registries, or indeed, a higher likelihood that a patient with comorbidities could also be examined for breast cancer.

Confounding by lifestyle factors (obesity, smoking, alcohol consumption) both increase breast cancer risk (Thun *et al*, 1997; Sonnenschein *et al*, 1999; McDonald *et al*, 2002), and contribute to general health deterioration, likely to impact independently on breast cancer prognosis (Sjol *et al*, 2003). Obesity, for example, is a risk factor for post-menopausal breast cancer (approximately 75% of cases) (Sonnenschein *et al*, 1999) and can exacerbate breast cancer prognosis (Bastarrachea *et al*, 1994; Abrahamson *et al*, 2006; Carmichael, 2006). Comparable to trends in other western countries (Boniface, 2006), obesity prevalence increased in Denmark by 7.1% from 1987 to 2001, particularly after 1998 (Sjol *et al*, 2003; Bendixen *et al*, 2004) and overweight prevalence in women increased by 10% (Bendixen *et al*, 2004). Furthermore, alcohol consumption (wine and hard liquor) and smoking have increased in Denmark over the past 10–20 years (Statistics Denmark, 2006). Consumption of alcohol before breast cancer diagnosis is thought to intimate a poorer prognosis (Thun *et al*, 1997; McDonald *et al*, 2002), but findings are inconsistent (Ewertz, 1993). The timing of the increased prevalence of obesity, alcohol consumption, and smoking corresponds with, and may, therefore, have impacted on survival among patients with severe comorbidities.

The main strengths of our study are its large size, the uniformly organised health-care system facilitating a population-based design with accurate survival estimates, reduced selection bias, and complete follow-up. Our study has some limitations: it may be prone to incidence-prevalence bias. Prevalent comorbid disease includes cases with long-term survival, who therefore have better survival than incident cases, in whom the full severity of comorbidity is represented (Goldman *et al*, 1983). To minimise such bias, we included comorbidity history recorded up to 10 years before breast cancer diagnosis. We used the Charlson comorbidity index to measure comorbidity, which has been used to assess the impact of comorbidities in many disease settings (Greenfield *et al*, 1993; Newschaffer *et al*, 1997; Horton *et al*, 2001; Mandelblatt *et al*, 2001). The Charlson index has some limitations, however: (a) it does not incorporate a measure of function (Greenfield *et al*, 1987); patients with identical scores can vary in comorbid disease severity, for example, manageable diabetes is classified within the same category as severely debilitating chronic obstructive pulmonary disease (Charlson score ‘1’). (b) The Charlson index is based on discharge diagnoses, which may not be entirely accurate. (c) The registration of comorbidities may have changed over the years, likely towards more complete registration.

In conclusion, we found a negative impact of comorbidity on breast cancer mortality. As breast cancer is the most commonly diagnosed cancer among women and the fifth most common cause of cancer-related death in women (Parkin *et al*, 2005), our observed increase in mortality among patients with severe comorbidity is of clinical and public health concern.

## Figures and Tables

**Table 1:** Number and percentage distribution of breast cancer patients by Charlson comorbidity score diagnosed in three time periods in Denmark

	**Charlson score**			
	**0**	**1–2**	**3+**	**Total**
*1995–1997*	2423	360	36	2819
	86%	13%	1%	100%
Median age in years (1995–1997)	59	73	72	
County of residence				
North Jutland	758 (84%)	129 (14%)	16 (2%)	903
Aarhus	954 (86%)	138 (13%)	14 (1%)	1106
Viborg	319 (84%)	60 (16%)	3 (1%)	382
Ringkojbing	392 (92%)	33 (8%)	3 (1%)	428
				
*1998–2000*	2518	431	54	3003
	84%	14%	2%	100%
Median age in years (1998–2000)	60	72	73	
County of residence				
North Jutland	818 (82%)	158 (16%)	24 (2%)	1000
Aarhus	935 (87%)	131 (12%)	12 (1%)	1078
Viborg	329 (79%)	73 (18%)	13 (3%)	415
Ringkojbing	436 (86%)	69 (14%)	5 (1%)	510
				
*2001–2004*	2829	565	84	3478
	81%	16%	2%	100%
Median age in years (2001–2004)	59	72	76	
County of residence				
North Jutland	903 (81%)	184 (16%)	33 (3%)	1120
Aarhus	1069 (82%)	207 (16%)	26 (2%)	1302
Viborg	410 (81%)	82 (16%)	13 (3%)	505
Ringkojbing	447 (81%)	92 (17%)	12 (2%)	551
				
Total	7770	1356	174	9300

Percentages have been rounded to the nearest decimal place and therefore may not sum to 100%.

**Table 2:** One-year and five-year survival after breast cancer for three comorbidity groups together with 1- and 5-year relative mortality for patients with Charlson scores of 1–2 and 3+ compared to patients with a Charlson score of 0 for each diagnostic period

	**Charlson score**		
	**0**	**1–2**	**3+**
*Breast cancer 1995–1997*			
Number of patients	2423	360	36
Median age (years)	59	73	72
1 year			
% Survival	93% (91–94%)	79% (74–83%)	72% (55–84%)
Crude MR	1 (reference)	3.15 (2.41–4.13)	4.23 (2.24–8.00)
MR adjusted for age	1 (reference)	2.43 (1.84–3.21)	3.21 (1.69–6.08)
5 year			
% Survival	71% (70–73%)	46% (41–51%)	39% (23–54%)
Crude MR	1 (reference)	2.38 (2.03–2.79)	3.09 (2.02–4.72)
MR adjusted for age	1 (reference)	1.89 (1.61–2.23)	2.43 (1.59–3.72)
			
*Breast cancer 1998–2000*			
Number of patients	2518	431	54
Median age (years)	60	72	73
1 year			
% Survival	93% (92–94%)	81% (77–85%)	80% (66–88%)
Crude MR	1 (reference)	2.74 (2.11–3.56)	3.07 (1.67–5.63)
MR adjusted for age	1 (reference)	2.01 (1.53–2.63)	2.25 (1.22–4.15)
5 year			
% Survival	74% (72–75%)	54% (49–58%)	38% (24–51%)
Crude MR	1 (reference)	2.10 (1.79–2.47)	3.15 (2.22–4.48)
MR adjusted for age	1 (reference)	1.60 (1.36–1.88)	2.34 (1.65–3.33)
			
*Breast cancer 2001–2004* a			
Number of patients	2829	565	84
Median age (years)	59	72	76
1 year			
% Survival	94% (93–95%)	85% (81–87%)	67% (55–76%)
Crude MR	1 (reference)	2.66 (2.06–3.44)	6.22 (4.17–9.27)
MR adjusted for age	1 (reference)	1.76 (1.35–2.30)	3.78 (2.51–5.68)

MR=mortality ratio.

Figures in parentheses show 95% confidence intervals.

a It was not possible to compute a 5-year survival or mortality ratio for patients diagnosed between 2001 and 2004 because of the short follow-up period.

**Table 3:** One-year and five-year survival for breast cancer patients in Denmark for three diagnostic periods

	**Diagnostic year**		
**Breast cancer**	**1995–1997**	**1998–2000**	**2001–2004**
Number of patients	2819	3003	3478
Median age (years)	61	62	62
			
*1 year*			
% Survival	91% (89–92%)	91% (90–92%)	92% (91–93%)
Crude MR	1 (reference)	0.98 (0.83–1.16)	0.88 (0.75–1.04)
Age adjusted MR	1 (reference)	0.95 (0.80–1.12)	0.86 (0.73–1.02)
MR adjusted for age and comorbidity	1 (reference)	0.92 (0.78–1.09)	0.80 (0.68–0.95)
			
*5 year*			
% Survival	68% (66–69%)	70% (68–72%)	—
Crude MR	1 (reference)	0.92 (0.84–1.01)	—
Age adjusted MR	1 (reference)	0.90 (0.82–0.99)	—
MR adjusted for age and comorbidity	1 (reference)	0.88 (0.80–0.97)	—

MR=mortality ratio.

Figures in parentheses indicate 95% confidence intervals.

**Table 4:** One-year and five-year survival among breast cancer patients with available stage information (*n*=7702) in Denmark for three diagnostic periods

	**Charlson score**		
	**0**	**1–2**	**3+**
*Breast cancer 1995–1997*	2225	281	29
Stage			
Local	1077 (48%)	125 (44%)	14 (48%)
Regional	962 (39%)	88 (31%)	6 (21%)
Distant	154 (7%)	23 (8%)	4 (14%)
Unstaged	132 (6%)	45 (16%)	5 (17%)
1-year			
% Survival	94% (93–95%)	81% (76–86%)	72% (52–85%)
Crude MR	1 (reference)	3.45 (2.50–4.75)	5.45 (2.67–11.14)
MR adjusted for age and stage	1 (reference)	2.67 (1.94–3.72)	3.33 (1.62–6.87)
5-year			
% Survival	73% (71–75%)	48% (42–53%)	45% (27–62%)
Crude MR	1 (reference)	2.44 (2.04–2.93)	2.92 (1.78–4.79)
MR adjusted for age and stage	1 (reference)	1.92 (1.59–2.32)	2.58 (1.56–4.25)
			
*Breast cancer 1998–2000*	2399	301	42
Stage			
Local	1149 (48%)	130 (43%)	25 (60%)
Regional	998 (42%)	105 (34%)	9 (21%)
Distant	138 (6%)	27 (9%)	1 (2%)
Unstaged	114 (5%)	43 (14%)	7 (17%)
1-year			
% Survival	94% (93–95%)	83% (78–86%)	81% (66–90%)
Crude MR	1 (reference)	3.22 (2.35–4.42)	3.61 (1.77–7.36)
MR adjusted for age and stage	1 (reference)	2.14 (1.54–2.96)	4.40 (2.11–9.14)
5-year			
% Survival	75% (74–77%)	54% (48–60%)	40% (25–54%)
Crude MR	1 (reference)	2.27 (1.88–2.73)	3.44 (2.30–5.13)
MR adjusted for age and stage	1 (reference)	1.63 (1.35–1.98)	3.78 (2.51–5.69)
			
*Breast cancer 2001–2004*	2566	357	52
Stage			
Local	1133 (44%)	148 (41%)	19 (37%)
Regional	1170 (46%)	133 (37%)	24 (46%)
Distant	114 (4%)	23 (6%)	3 (6%)
Unstaged	149 (6%)	53 (15%)	6 (12%)
1-year			
% Survival	95% (94–96%)	87% (83–90%)	71% (57–81%)
Crude MR	1 (reference)	2.80 (2.01–3.89)	6.43 (3.77–10.97)
MR adjusted for age and stage	1 (reference)	1.91 (1.36–2.69)	4.45 (2.58–7.66)

MR=mortality ratio.

Figures in parentheses indicate 95% confidence intervals.
